# Improved ICU design reduces acquisition of antibiotic-resistant bacteria: a quasi-experimental observational study

**DOI:** 10.1186/cc10446

**Published:** 2011-09-14

**Authors:** Phillip D Levin, Mila Golovanevski, Allon E Moses, Charles L Sprung, Shmuel Benenson

**Affiliations:** 1 Department of Anesthesia and Critical Care, Hadassah-Hebrew University Medical Center, POB 12000, Jerusalem 91120, Israel; 2 Department of Clinical Microbiology and Infectious Disease, Hadassah-Hebrew University Medical Center, POB 12000, Jerusalem 91120, Israel

## Abstract

**Introduction:**

The role of ICU design and particularly single-patient rooms in decreasing bacterial transmission between ICU patients has been debated. A recent change in our ICU allowed further investigation.

**Methods:**

Pre-move ICU-A and pre-move ICU-B were open-plan units. In March 2007, ICU-A moved to single-patient rooms (post-move ICU-A). ICU-B remained unchanged (post-move ICU-B). The same physicians cover both ICUs. Cultures of specified resistant organisms in surveillance or clinical cultures from consecutive patients staying >48 hours were compared for the different ICUs and periods to assess the effect of ICU design on acquisition of resistant organisms.

**Results:**

Data were collected for 62, 62, 44 and 39 patients from pre-move ICU-A, post-move ICU-A, pre-move ICU-B and post-move ICU-B, respectively. Fewer post-move ICU-A patients acquired resistant organisms (3/62, 5%) compared with post-move ICU-B patients (7/39, 18%; *P *= 0.043, *P *= 0.011 using survival analysis) or pre-move ICU-A patients (14/62, 23%; *P *= 0.004, *P *= 0.012 on survival analysis). Only the admission period was significant for acquisition of resistant organisms comparing pre-move ICU-A with post-move ICU-A (hazard ratio = 5.18, 95% confidence interval = 1.03 to 16.06; *P *= 0.025). More antibiotic-free days were recorded in post-move ICU-A (median = 3, interquartile range = 0 to 5) versus post-move ICU-B (median = 0, interquartile range = 0 to 4; *P *= 0.070) or pre-move ICU-A (median = 0, interquartile range = 0 to 4; *P *= 0.017). Adequate hand hygiene was observed on 140/242 (58%) occasions in post-move ICU-A versus 23/66 (35%) occasions in post-move ICU-B (*P *< 0.001).

**Conclusions:**

Improved ICU design, and particularly use of single-patient rooms, decreases acquisition of resistant bacteria and antibiotic use. This observation should be considered in future ICU design.

## Introduction

The Centers for Disease Control estimate that annually in the US approximately 1.7 million patients suffer from hospital-acquired infections with 99,000 deaths [1] and a cost of up to $33.8 billion [2]. About 20% of ICU patients will develop nosocomial infections, often caused by resistant bacteria [3,4], and many more become colonized by resistant bacteria [4,5].

Bacteria are transmitted between ICU patients by direct contact (principally via caregivers' hands), droplets (for example, from infected airway secretions) and via fomites (inanimate objects in the ICU environment) [6,7]. Improved ICU architecture might limit the spread of bacteria. Single-patient rooms, for example, have been in use for almost a century [8] despite equivocal evidence [9].

A recent upgrade to one of our ICUs presented the opportunity to test the effect of improved architecture on acquisition of resistant bacteria. Prior to the upgrade, the ICUs were both open plan and existed in two separate locations. In March 2007, one location was refurbished, with each bed being placed in a separate closed room, while the other ICU remained unchanged. This situation presented the opportunity to compare the acquisition rates of resistant bacteria between patients admitted to a single-room ICU and to an open-plan ICU, with the single-room ICU acting as the intervention location and the open-plan ICU acting as a control environment.

## Materials and methods

### Study design

**Figure 1:**
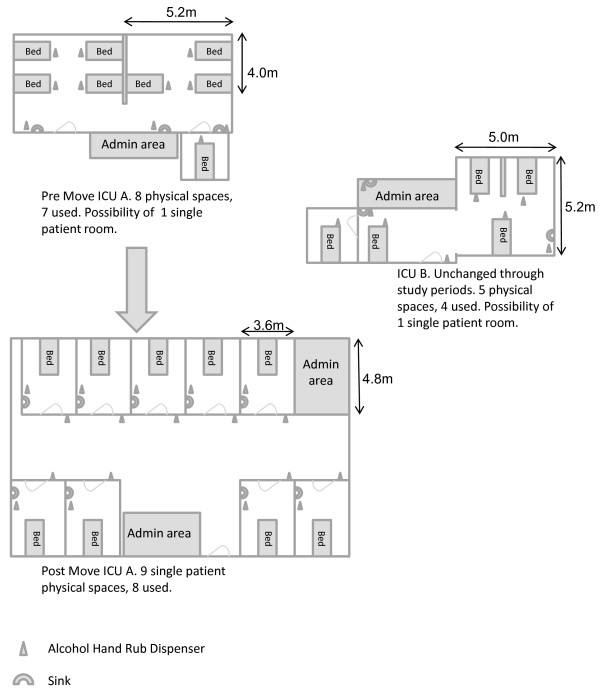
**ICU layouts and changes**.

The study was performed in the general ICU of the Hadassah Hebrew University Medical Center, Jerusalem, Israel, a 750-bed urban academic tertiary referral center. The ICU exists in two separate locations. Prior to March 2007 these two sites were open plan (ICU-A, seven open-plan beds; ICU-B, four beds with dividers). In March 2007 ICU-A moved to a new location with eight beds each in a separate closed room, while ICU-B remained unchanged (Figure 1). Patient location is determined by space availability. Each ICU had a separate nursing staff, but patients were treated by the same team of physicians.

Data were collected prospectively from all patients admitted for longer than 48 hours. July 2005 to January 2006 comprised the pre-move period, while October 2007 to February 2008 was the post-move period. Patients transferred from ICU-A to ICU-B or *vice versa *and ICU patients admitted to the post-anesthesia recovery unit were excluded. The Hadassah Hebrew University Medical Center ethics committee approved the study (number 1-20/05/01) and waived any requirement for informed consent prior to data collection.

All patients were treated with standard infection control precautions. Alcohol-based hand rub was freely available next to each bed pre-move and next to each bed or at the entrance to each room post-move (Figure 1). In post-move ICU-A, each room had its own sink, alcohol-based hand rub dispenser, computer and basic medical supplies (syringes, needles, suction equipment, and so forth). In pre-move ICU-A and in both pre-move and post-move ICU-B, computers and sinks were shared and medical equipment was available on carts common to all patients. The nurse to patient ratio was 1:2 in both ICUs and at both time points.

Data collection included the ICU and period of admission, demographics, admission functional status (defined as functionally independent or not), outcome, Acute Physiology and Chronic Health Evaluation II score (for first 24 hours) [10], antibiotic administration, occurrence of nosocomial infection and results of all microbiological studies (both surveillance and clinical).

### Nosocomial acquisition of resistant bacteria

Data were collected on methicillin-resistant *Staphylococcus aureus *(MRSA), vancomycin-resistant enterococci, *Acinetobacter baumannii *resistant to ceftazidime, carbapenem-resistant Enterobacteriaceae, and extended-spectrum β-lactamase-producing Enterobacteriaceae (defined as Enterobacteriaceae resistant to ceftazidime). Surveillance cultures (from the nose, axilla and perineum) were performed sporadically in the pre-move ICUs and on ICU admission and once weekly in both ICUs during the post-move period. Surveillance was performed for MRSA, vancomycin-resistant enterococci, carbapenem-resistant *Enterobacteriaceae *and *A. baumannii*.

Hospital cultures performed during the 6 months prior to ICU admission were also assessed. A positive culture for one of the index bacteria during the 6 months prior to ICU admission or during the first 48 hours of ICU admission defined the patient as colonized on admission. A positive culture appearing after the first 48 hours or up to 48 hours after ICU discharge indicated colonization/infection that occurred during the ICU stay. Patients who were colonized/infected with any of the defined resistant bacteria on ICU admission could not become colonized/infected with that bacterial species during their ICU stay and were excluded from analysis regarding the risk of acquiring the specific resistant bacteria in the ICU. Acquisition of other resistant bacteria remained possible.

A composite measure representing colonization/infection by any of the index bacteria was defined as follows. A patient in whom any of the defined organisms were cultured within 48 hours of ICU admission met the criteria for the composite measure of any resistant organism on admission. A patient for whom any of the defined organisms were discovered in cultures taken after 48 hours in the ICU, and who was not infected/colonized with the specific organism on admission, met the criteria for the composite measure of ICU acquisition of any resistant organism.

### Hand hygiene compliance

Hand hygiene compliance was observed covertly by a single trained observer during the post-move period based on criteria defined by the World Health Organization five moments of hand hygiene [11]. Hand hygiene observations were performed during office hours and data were collected separately for physicians and nurses. Data were collected on at least 40 hand hygiene opportunities per month per ICU. Adequate hand hygiene was considered use of either alcohol hand rub or 4% chlorhexidine soap and water. The proportion of the total number of observed hand hygiene opportunities where adequate hand hygiene was performed was recorded.

### Antibiotic use

Use of all antibiotics was recorded. To detect a possible decrease in antibiotic usage, the median number (plus interquartile range) of antibiotic-free days for ICU-A and ICU-B before and after the ICU-A move was calculated and compared.

### Nosocomial infections

The presence of nosocomial bloodstream infections, pneumonia or urinary tract infection was recorded based on Centers for Disease Control definitions [12]. The presence of nosocomial infection was determined prospectively throughout the study by a dedicated study nurse/physician based on real-time review of chest X-ray scans, blood and urine cultures, and the patients' clinical courses. Training and an occasional audit were performed by the principal investigators. Borderline cases and uncertainty were also resolved by the principal investigators. The proportion of patients developing one of these infections after the first 48 hours in the ICU was compared.

### Data analysis

The primary hypothesis was that admission to post-move ICU-A decreased the risk of an individual patient acquiring resistant bacteria in the ICU. To test this hypothesis, the proportion of patients acquiring defined resistant bacteria was compared across the different ICUs and time periods. The primary comparison was between post-move ICU-A and post-move ICU-B, representing the intervention and contemporary control groups. Comparisons were also performed between post-move ICU-A and pre-move ICU-A (to demonstrate a change over time), between pre-move ICU-A and pre-move ICU-B (to ascertain similarity), and finally between pre-move ICU-B and post-move ICU-B (to demonstrate consistency).

Risk for acquisition of resistant organisms is partly dependent on colonization pressure [13-15]. The total number of patient-days during which an uninfected/uncolonized patient was exposed to other patients who were infected/colonized with a resistant organism was therefore calculated. This figure represented the colonization pressure to which each individual patient was subjected. For example, Patient X is a new patient uncolonized with resistant organisms who has been admitted to the ICU for 5 days. A culture taken on day 5 is positive for a resistant organism. During the 5 days prior to the discovery of a resistant organism there were three other patients present in the ICU with a resistant organism. Patient X was therefore exposed to a colonization pressure of 5 × 3 = 15.

The risk of acquiring a resistant organism was examined using univariate analysis and multivariate Cox regression analysis. Variables from the univariate analysis were included in the multivariate analysis if significant at *P *≤0.05. Proportions were compared using the chi-squared test or Pearson's exact test. Normally distributed continuous variables were compared using Student's *t *test; otherwise, the Wilcoxon test was used. Data collection periods were determined by available resources. Statistical significance was defined as two-tailed *P *< 0.05. SAS version 8.02 (SAS Corporation, Cary, NC, USA) was used for all data analysis.

## Results

Data were collected for 207 patients, including 62 pre-move ICU-A patients, 62 post-move ICU-A patients, 44 pre-move ICU-B patients and 39 post-move ICU-B patients. An additional 33 ICU patients were excluded due to admission to the post-anesthesia care unit (eight prior to the ICU move and 12 following the move) or transfer between ICU-A and ICU-B (four before the move, nine after). Demographics, medical history and outcome data are shown in Table 1.

**Table 1:** Admission and outcome variables

	Pre-A (*n *= 62)	Pre-B (*n *= 44)	Post-A (*n *= 62)	Post-B (*n *= 39)	Pre-A vs. pre-B	Post-A vs. post-B	Pre-A vs. post-A	Pre-B vs. post-B
Demographics								
Age (years)	57 ± 24	59 ± 22	57 ± 23	53 ± 24	0.609	0.418	0.939	0.242
Gender (male)	36 (58)	29 (66)	40 (65)	32 (82)	0.414	0.058	0.461	0.096
APACHE II score	21 ± 9	19 ± 8	18 ± 9	17 ± 8	0.144	0.684	0.028	0.321
Pre-hospital functionally independent	45 (73)	33 (75)	47 (76)	32 (82)	0.781	0.621	0.682	0.437
Antibiotic therapy prior to ICU admission (days)	37 (60)	16 (36)	20 (32)	18 (46)	0.008	0.161	0.001	0.365
Hospital admission prior to ICU admission (days)	37 (60)	23 (62)	21 (34)	20 (51)	0.449	0.083	0.004	0.928
Trauma ICU admission etiology	14 (23)	8 (18)	16 (26)	9 (23)	0.582	0.757	0.675	0.581
Surgery prior to ICU admission	25 (40)	29 (66)	24 (39)	22 (56)	0.009	0.082	0.854	0.375
Past medical history								
Ischemic heart disease^a^	9 (15)	2 (5)	4 (6)	1 (3)	0.117	0.646	0.143	1.000
Respiratory disease^a^	2 (3)	0 (0)	2 (3)	0 (0)	0.510	0.521	1.000	-
Diabetes mellitus	17 (27)	16 (36)	9 (15)	11 (28)	0.327	0.093	0.078	0.773
Chronic steroid use	8 (13)	5 (11)	4 (6)	4 (10)	0.812	0.491	0.224	1.000
Cirrhosis^a^	3 (5)	2 (5)	4 (6)	1 (3)	0.944	0.381	1.000	1.000
Chronic dialysis^a^	4 (6)	1 (2)	2 (3)	2 (5)	0.400	0.639	0.677	0.598
Solid organ transplant	1 (2)	0 (0)	3 (5)	1 (3)	1.000	1.000	0.619	0.470
Immunosuppressed^a^	3 (5)	2 (5)	4 (6)	4 (10)	1.000	0.707	1.000	0.412
Malignant disease	16 (26)	9 (20)	12 (19)	6 (15)	0.523	0.791	0.390	0.549
Colonization pressure (mean daily)	11.4 ± 22.2	10.0 ± 14.9	8.5 ± 11	5.2 ± 6	0.706	0.052	0.362	0.052
Outcomes								
Do-not-resuscitate order given	7 (11)	5 (11)	7 (11)	1 (3)	1.000	0.147	1.000	0.207
ICU length of stay (days)	8.5 (5 to 15)	11 (5 to 16)	8 (4 to 15)	7 (4 to 14)	0.662	0.477	0.442	0.065
Hospital length of stay (days)	25 (16 to 47)	32.5 (16 to 50)	26 (16 to 42)	27 (15 to 45)	0.600	0.878	0.677	0.350
ICU mortality	10 (16)	5 (11)	10 (16)	6 (15)	0.488	1.000	1.000	0.590

Data presented as mean ± standard deviation, *n *(%), *n *(interquartile range) or *P *value. Pre-A, pre-move ICU-A; pre-B, pre-move ICU-B; post-A, post-move ICU-A; post-B, post-move ICU-B. ^a^As defined in the Acute Physiology and Chronic Health Evaluation (APACHE) II score [10].

**Figure 2:**
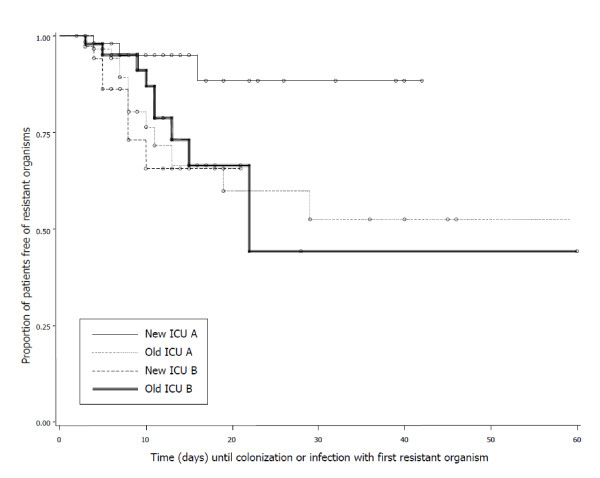
**Time course for acquisition of resistant organisms in the ICU**.

The ICU admission prevalence and the ICU acquisition of resistant bacteria are shown in Table 2. Overall, the admission prevalence of resistant bacteria was higher in the pre-move ICUs versus the post-move ICUs: 38/106 patients (36%, 95% confidence interval (CI) = 27 to 45%) versus 18/101 patients (18%, 95% CI = 10 to 25%) (*P *= 0.004). The time course for acquisition of resistant organisms is shown in Figure 2 and is analyzed below.

**Table 2:** Prevalence of resistant bacteria on admission and incidence of resistant bacteria acquisition during ICU stay

	Pre-A	Pre-B	Pre-A vs. pre-B	Post-A	Post-B	Post-A vs. post-B
**Prevalence of resistant bacteria on admission**						
*Acinetobacter baumannii*^a^	11/62 (18)	6/44 (14)	0.570	5/62 (8)	1/39 (3)	0.401
Carbapenem-resistant Enterobacteriaceae	0/62 (0)	0/44 (0)	-	4/62 (6)	1/39 (3)	0.646
ESBL Enterobacteriaceae^a^	11/62 (18)	6/44 (14)	0.570	8/62 (13)	3/39 (8)	0.523
MRSA	5/62 (8)	4/44 (9)	1.000	2/62 (3)	0/39 (0)	0.521
VRE	5/62 (8)	1/44 (2)	0.397	0/62 (0)	3/39 (8)	0.055
Any resistant organism	25/62 (40)	13/44 (30)	0.253	11/62 (18)	7/39 (18)	0.979
**Acquisition of resistant bacteria during ICU stay**						
*Acinetobacter baumannii*^a^	8/51 (16)	5/38 (13)	0.738	1/57 (2)	3/38 (8)	0.298
Carbapenem-resistant Enterobacteriaceae	0/62 (0)	1/44 (2)	0.415	0/58 (0)	2/38 (5)	0.154
ESBL Enterobacteriaceae^a^	4/51 (8)	2/38 (5)	1.000	1/54 (2)	0/36 (0)	1.000
MRSA	3/57 (5)	2/40 (5)	1.000	0/60 (0)	3/39 (8)	0.058
VRE	1/57 (2)	0/43 (0)	1.000	1/62 (2)	0/36 (0)	1.000
Any resistant organism	14/62 (23)	9/44 (20)	0.794	3/62 (5)	7/39 (18)	0.043

Data presented as number of positive patients/patients at risk (%) or *P *value. The denominator for admission prevalence is the total number of study patients included. The denominator for ICU acquisition is determined by number of included patients minus patients with resistant bacteria on admission. As no patients were colonized/infected with all five resistant organisms on admission, the denominator for ICU acquisition of any of the resistant organisms remained equivalent to the total study population. ESBL, extended spectrum β-lactamase; MRSA, methicillin-resistant *Staphylococcus aureus*; VRE, vancomycin-resistant enterococci. pre-A, pre-move ICU-A; pre-B, pre-move ICU-B; post-A, post-move ICU-A; post-B, post-move ICU-B;. ^a^Resistant to at least ceftazidime.

### Principal comparison: post-move ICU-A versus post-move ICU-B

The patient populations in the single-room post-move ICU-A and the contemporary open-plan post-move ICU-B were similar, with no demographic or past medical history variables being significantly different. The admission prevalence of resistant bacteria was equal between the two ICUs.

Significantly fewer post-move ICU-A patients (3/62, 5%; 95% CI = 0 to 10%) acquired a resistant organism during their ICU stay when compared with post-move ICU-B patients (7/39, 18%; 95% CI = 6 to 30%; *P *= 0.043), representing a decrease of 72%. This was confirmed using time-course analysis (*P *= 0.011) and occurred despite a higher colonization pressure in post-move ICU-A (8.5 ± 11 vs. 5.2 ± 6 days/patient, *P *= 0.052). Using multivariate Cox regression (including colonization pressure and ICU of admission), only the admitting ICU was a significant predictor for acquisition of resistant organisms (post-move ICU-B vs. post-move ICU-A: hazard ratio = 4.07, 95% CI = 1.03 to 16.1; *P *= 0.045). There was also a trend to more antibiotic-free days in post-move ICU-A (median = 3, interquartile range (IQR) = 0 to 5 vs. median = 0, IQR = 0 to 4; *P *= 0.070).

Regarding hand hygiene practice, physicians (who covered both ICUs) were compliant on 140/242 occasions (58%, 95% CI = 52 to 64%) in post-move ICU-A versus 23/66 occasions (35%, 95% CI = 23 to 46%) in post-move ICU-B (*P *< 0.001). Nurses, however, complied on 129/188 occasions (69%, 95% CI = 62 to 75%) in post-move ICU-A versus 118/206 occasions (57%, 95% CI = 50 to 64%) in post-move ICU-B (*P *= 0.020).

### Assessing change: post-move ICU-A versus pre-move ICU-A

Significantly fewer post-move ICU-A patients acquired a resistant organism compared with pre-move ICU-A patients (3/62, 5%; 95% CI = 0 to 10% vs. 14/62, 23%; 95% CI = 12 to 33%; *P *= 0.004, *P *= 0.012 on time-course analysis) (Figure 2). There were significantly more antibiotic-free days in post-move ICU-A (median = 3, IQR = 0 to 5 vs. median = 0, IQR = 0 to 4 for pre-move ICU-A; *P *= 0.017).

The Acute Physiology and Chronic Health Evaluation II score, antibiotic therapy prior to ICU admission, hospital admission prior to ICU admission and prevalence of resistant bacteria on ICU admission were significantly higher or more frequent in pre-move ICU-A. Only the ICU of admission was a significant predictor of acquisition of a resistant organism on multivariate analysis (pre-move ICU-A vs. post-move ICU-A: OR 5.18, 95% CI = 1.03 to 16.06; *P *= 0.025). Colonization pressure was also higher in pre-move ICU-A (11.4 ± 22 vs. 8.5 ± 11 for post-move ICU-A), but the difference did not reach statistical significance (*P *= 0.362) and was therefore not included in the multivariate analysis.

### Baseline comparison: pre-move ICU-A versus pre-move ICU-B

These patient populations were similar with the exception that surgery prior to ICU admission was more common and preadmission antibiotic use less frequent in pre-move ICU-B patients (Table 1). There were no significant differences in the admission prevalence or ICU acquisition of resistant bacteria (Table 1 and Figure 2; *P *= 0.615). The number of antibiotic-free days was equivalent (pre-move ICU-A vs. pre-move ICU-B: median antibiotic-free days = 0, IQR = 0 to 4 vs. median = 1, IQR = 0 to 3; *P *= 0.515).

### Consistency: pre-move ICU-B versus post-move ICU-B

There were no differences in patient population, acquisition of resistant bacteria (post-move ICU-B vs. pre-move ICU-B patients: 7/39, 18%; 95% CI = 6 to 30% vs. 9/44, 20%; 95% CI = 8 to 32%; *P *= 0.773, *P *= 0.347 by time-course analysis) or number of antibiotic-free days (post-move ICU-B vs. pre-move ICU-B: median = 0, IQR = 0 to 4 vs. median = 1, IQR = 0 to 3; *P *= 0.717), although the colonization pressure was nonsignificantly higher in pre-move ICU-B (Table 1).

### Nosocomial infections

The proportions of patients who developed nosocomial bloodstream infections, pneumonia or urinary tract infection over the study periods are shown in Table 3. Overall there was a decrease in the occurrence of nosocomial infections from the pre-move to post-move periods. However, there was no difference in the incidence rate when comparing ICU-A with ICU-B within each time period.

**Table 3:** Comparison of nosocomial infection rates for pre-move and post-move ICU-A and pre-move and post-move ICU-B

	Pre-A (*n *= 62)	Pre-B (*n *= 44)	Post-A (*n *= 62)	Post-B (*n *= 39)	Pre-A vs. pre-B	Post-A vs. post-B	Pre-A vs. post-A	Pre-B vs. post-B
Pneumonia	13 (21)	12 (27)	5 (8)	5 (13)	0.45	0.50	0.04	0.12
Bacteremia	14 (23)	13 (13)	7 (11)	3 (8)	0.42	0.74	0.09	0.01
UTI	7 (11)	3 (7)	8 (13)	1 (3)	0.52	0.15	0.78	0.62

Data presented as *n *(%) or *P *value. Pre-A, pre-move ICU-A; pre-B, pre-move ICU-B; post-A, post-move ICU-A; post-B, post-move ICU-B; UTI, urinary tract infection.

## Discussion

Improved ICU architecture, including a change to single rooms, led to a statistically and clinically significant 72% decrease in the risk of acquiring resistant bacteria and a decrease in antibiotic usage. The strength of these findings lies in the comparison of patients admitted at the same time to two separate ICU environments, one open plan and the other single rooms, and treated by the same physician team. These findings have important implications for future ICU design.

The precise element of the design change that contributed to the decrease in acquisition of resistant bacteria cannot be determined, as the design change included many elements - single rooms, increased patient area, increased number of sinks per patient, and so forth. Each of these individual elements may have contributed to improved infection control [16]. Further, hand hygiene compliance (particularly by physicians) was higher in the single-room ICU, possibly indicating that ICU design may have an effect on staff behavior and not only on the physical environment.

Investigating the effect of ICU design on acquisition of resistant bacteria is methodologically difficult. Costs prevent ICUs from being constructed for research purposes only, and alterations in ICU design involve a range of structural changes that could affect spread of resistant bacteria. Further, most studies examining the effect of changes in ICU design are of a before/after design, or are performed in the context of an outbreak of resistant organisms and involve multiple infection control measures, meaning that the contribution of ICU design alone is impossible to determine [9]. As before/after studies are conducted over long time periods, the effects of general environmental (for example, antibiotic flora) and educational (for example, infection control awareness) changes are also difficult to account for. The current study collected contemporary data in parallel from two branches of the same ICU covered by the same physician. This comparison counteracted many of these limitations and allowed investigation of the effect of change in ICU design in isolation.

Two recent studies have addressed the issue of single-patient ICU rooms: a Canadian observational trial of an ICU move that led to a decrease of approximately 50% in ICU acquisition of certain organisms [17], and an interventional interrupted time-series study examining the ICU spread of MRSA that showed no benefit from isolation rooms [18]. In the MRSA study, patients were transferred to isolation rooms only after identification of MRSA (a process taking approximately 3 days), the single rooms were within an open-plan ICU (rather than a unit comprising single rooms only), and hand hygiene practices did not change over the study period [18].

Both our study and the Canadian observational study [17] addressed these issues. Patients were admitted to single rooms at the outset, potentially protecting them from colonization with resistant bacteria from other undetected carriers. Second, the study ICUs comprised exclusively single rooms, reducing patient exposure to equipment common to multiple patients. Inanimate objects (such as computer equipment, sink faucets, beds and chairs) become colonized with resistant bacteria [19-21] and have been implicated in their transfer between patients [22]. The use of single rooms within an open-plan unit might thus be inadequate as the environment around the single room might still remain a reservoir for transmission of bacteria, while separation of patients and their equipment may provide additional benefit. Third, our study suggests that the effect of ICU design may be mediated in part by behavioral change. Hand hygiene for the same physician team was better in the single-room ICU than in the open-plan ICU. This was not examined in the Canadian observational trial and suggests that ICU design has effects beyond the ergonomic fostering improved infection control awareness, once again potentially limiting the effectiveness of patient isolation within an open-plan unit.

The present study has several limitations. First, the study was small, had a short follow-up period and did not show an effect on mortality or ICU length of stay. Second, routine surveillance for resistant bacteria was performed only in the post-move ICUs, which might be seen as a confounder in the comparison of the pre-move and post-move ICUs. However, the main outcome measure was a comparison of post-move ICU-A with the control patients in post-move ICU-B, where equivalent surveillance culturing was performed. Further, increased surveillance culturing would be expected to increase detection of resistant bacteria. Despite this, acquisition in post-move ICU-A was lower than that in pre-move ICU-A, strengthening the study findings. The changes found over time in fact emphasize the necessity for contemporary controls when assessing infection control interventions. Finally, patient distribution between the two ICUs was not truly random. However, as it was rare that more than one ICU bed was available at a specific time, allocation of the bed location was not determined by physician preference, but rather by availability. Further, the admission prevalence of resistant bacteria was no different when comparing pre-move ICU-A to pre-move ICU-B or post-move ICU-A to post-move ICU-B.

The American Institute of Architects recommends single-patient rooms in their guidelines for hospital design [23], while in France single-patient rooms have been required in all new hospitals constructed over the past 20 years [24]. These interventions cost millions of dollars. Despite this cost, data supporting the use of single-patient rooms have been limited. Our study contributes a high-quality, quasi-experimental comparison of open-space versus single-room ICU accommodation and shows that improved ICU design reduces the acquisition of resistant bacteria and use of antibiotics.

## Conclusions

The use of single rooms to prevent cross-infection/colonization between ICU patients is common but expensive and unproven. This investigation compared acquisition of resistant bacteria and antibiotic use between ICU patients admitted to single rooms and contemporary patients admitted to a open-plan ICU. The study showed a 72% decrease in colonization/infection by resistant bacteria for patients in single rooms and an increase in antibiotic-free days. The present study may influence the way ICUs are designed in the future.

## Key messages

• This is a prospective observational examination of the effect of ICU design on the acquisition of resistant bacteria including patients in a single-room ICU and comparing them with patients admitted at the same time to an open-plan ICU, both ICUs being treated by the same physician team.

• The improved ICU design (principally the use of single-patient rooms) significantly decreased acquisition of antibiotic-resistant bacteria by 72%.

• This change also led to an increased number of antibiotic-free days.

• Whether the decreased acquisition of bacteria resulted from the physical change in ICU design itself or reflected an effect of ICU design on staff conduct (through increased hand hygiene, for example) could not be determined.

## Abbreviations

CI: confidence interval; IQR: interquartile range; MRSA: methicillin-resistant *Staphylococcus aureus*;

## Competing interests

The authors declare that they have no competing interests.

## Authors' contributions

PDL was the principal investigator, responsible for study design, data analysis and manuscript preparation. MG performed data collection. AEM was responsible for the provision of microbiology data and study overview. CLS was the study mentor, and handled the study design, data analysis and manuscript review. SB handled the study design, data analysis and manuscript preparation. All authors read and approved the final manuscript.
